# Mechanisms of Action of EGFR Tyrosine Kinase Receptor Incorporated in Extracellular Vesicles

**DOI:** 10.3390/cells9112505

**Published:** 2020-11-19

**Authors:** Laura C. Zanetti-Domingues, Scott E. Bonner, Marisa L. Martin-Fernandez, Veronica Huber

**Affiliations:** 1 Central Laser Facility, Research Complex at Harwell, Rutherford Appleton Laboratory, Didcot OX11 0FA, UK; marisa.martin-fernandez@stfc.ac.uk; 2 The Wood Lab, Department of Paediatrics, University of Oxford, Oxford OX1 3QX, UK; scott.bonner@wolfson.ox.ac.uk; 3 Unit of Immunotherapy of Human Tumors, Fondazione IRCCS Istituto Nazionale dei Tumori, 20133 Milan, Italy

**Keywords:** Epidermal Growth Factor Receptor (EGFR), extracellular vesicles (EVs), ExTRAcrine signalling, EV heterogeneity, tumour microenvironment, microenvironment subversion, immune suppression, epithelial-to-mesenchymal transition (EMT), cancer, therapy resistance

## Abstract

EGFR and some of the cognate ligands extensively traffic in extracellular vesicles (EVs) from different biogenesis pathways. EGFR belongs to a family of four homologous tyrosine kinase receptors (TKRs). This family are one of the major drivers of cancer and is involved in several of the most frequent malignancies such as non-small cell lung cancer, breast cancer, colorectal cancer and ovarian cancer. The carrier EVs exert crucial biological effects on recipient cells, impacting immunity, pre-metastatic niche preparation, angiogenesis, cancer cell stemness and horizontal oncogene transfer. While EV-mediated EGFR signalling is important to EGFR-driven cancers, little is known about the precise mechanisms by which TKRs incorporated in EVs play their biological role, their stoichiometry and associations to other proteins relevant to cancer pathology and EV biogenesis, and their means of incorporation in the target cell. In addition, it remains unclear whether different subtypes of EVs incorporate different complexes of TKRs with specific functions. A raft of high spatial and temporal resolution methods is emerging that could solve these and other questions regarding the activity of EGFR and its ligands in EVs. More importantly, methods are emerging to block or mitigate EV activity to suppress cancer progression and drug resistance. By highlighting key findings and areas that remain obscure at the intersection of EGFR signalling and EV action, we hope to cross-fertilise the two fields and speed up the application of novel techniques and paradigms to both.

## 1. Introduction

Extracellular vesicles (EVs) are membranous nanoparticles of a wide range of sizes released from nearly all eukaryotic and prokaryotic cells [1,2,3]. EVs have been identified in a plethora of biological materials including blood, urine, faeces, bile, breast milk, as well as synovial, lacrimal, seminal, ascites and bronchoalveolar lavage fluids [4]. EVs are released naturally in response to cellular stimuli, as well as due to changes in pH, hypoxia, injury and other cellular stressors, and in response to complement activation [5,6,7,8]. While initially thought to be a mechanism of eliminating waste from cells [9], in 1996 Raposo et al. documented that EVs could stimulate adaptive immune responses [10]. From there, EVs became recognised as important mediators of intercellular communication through the bioactive transfer of their protein, lipid and nucleic acid cargoes to recipient cells [11,12,13,14]. 

Over the last few decades, EVs have been shown to mediate phenotypic alterations in physiological and pathological conditions. Most notably, the role of EVs in cancer development and progression, including their proposed involvement in metastatic growth and chemotherapeutic resistance, has been extensively studied. Along with active efflux mechanisms, EVs have been shown to aid removal of chemotherapeutics from cancer cells [15,16]. Furthermore, EVs have also been shown to be involved in tumour progression and metastasis by mediating epithelial-to-mesenchymal transition (EMT), migration, invasion, angiogenesis, immune modulation and reprogramming healthy cells to a cancerous phenotype in establishing a pre-metastatic niche [17,18].

The Epidermal Growth Factor Receptor (EGFR) family of receptor tyrosine kinases (RTKs) is a major player in a variety of epithelial malignancies, such as breast cancer, non-small cell lung cancer, colorectal, gastric and ovarian cancer, and glioblastoma. This has led to the development of targeted therapeutics, including tyrosine kinase inhibitors (TKIs) and antibodies, such as Herceptin, representing a milestone in cancer therapy [19].

The HER family includes four members—EGFR, Human Epidermal Growth Factor Receptor HER2, HER3 and HER4; their splicing variants; and 11 cognate ligands [20,21,22,23,24,25]. So far, EGFR, HER2 and some ligands have been found to be expressed on EVs, where they enhance the malignant potential of their parental neoplastic cells. 

The role of EGFR family proteins on cancer cells is well recognised and has been extensively reviewed (see Maennling et al. [26] for recent updates to the state-of-the-art), highlighting commonalities and specificities in their modes of action and mutational patterns between cancer types [27,28]. As of yet there is no comparable understanding of the roles of this receptor family at the EV level, even though EVs have emerged as a major driver of tumourigenesis and are exploited as biomarkers in liquid biopsies.

Given the biological and clinical relevance of EGFR and its ligands, herein we discuss key findings regarding their expression on EVs and the roles of these proteins in EV signalling and uptake. Additionally, we will shine a spotlight on outstanding questions regarding the relevance of EGFR expression on EVs in different types of cancer, their implications in disease progression, in respective therapeutic approaches and as biomarkers, and discuss what techniques and frameworks can be brought to bear on them.

## 2. Extracellular Vesicles and Their Biogenesis

The term “extracellular vesicles” encompasses a variety of different vesicle subclasses mainly based on their respective biogenesis and biophysical properties. The most extensively studied subtypes are exosomes and microvesicles (MVs).

**Figure 1:**
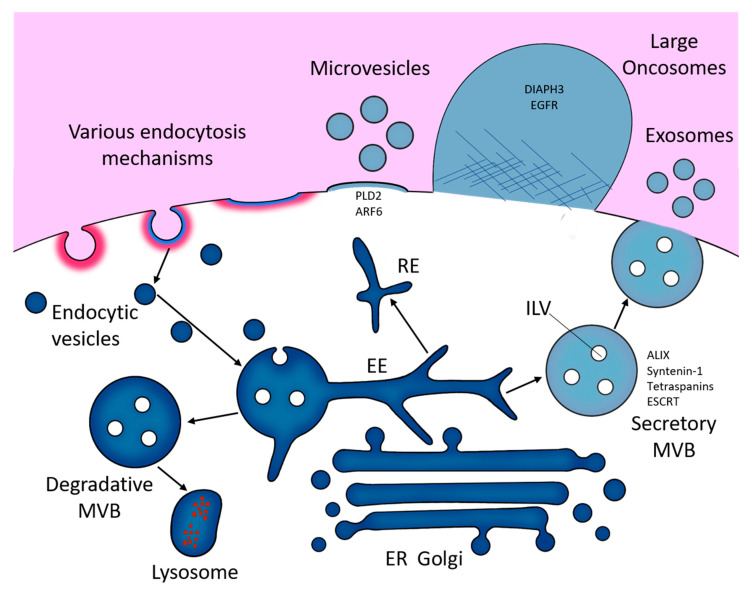
A diagram of the possible routes of formation of extracellular vesicles (EVs), via the endosomal pathway (exosomes), directly from membrane shedding (microvesicles) and via large-scale, non-apoptotic blebbing (large oncosomes). EE, early endosome; ER, endoplasmic reticulum; RE, recycling endosome.

Exosomes range in size from roughly 30 nm to 150 nm in diameter [29,30,31]. They are formed within the endolysosomal pathway (Figure 1). First, the inward budding of the cell membrane forms membranous vacuoles called endosomes. Subsequent inward invaginations of endosomal membranes forms intra-luminal vesicles (ILVs) which themselves are early exosomes. Through multiple inward invagination events, endosomes become filled with ILVs and become referred to as multivesicular bodies (MVBs) [32]. MVBs displaying specific surface proteins fuse with lysosomes to degrade ILV contents. These include RAB7A, HSP70-HSP90 organising protein (HOP) complexes and soluble *N*-ethylmaleimide-sensitive factor attachment protein receptor (SNARE) complexes, including vesicle-associated membrane protein 7 (VAMP7), and syntaxin 7 and 8 (STX7/8). Otherwise, MVBs traffic to and fuse with the cell membrane releasing their ILVs, then termed exosomes [33,34].

Exosomal formation in MVBs can occur dependently or independently of proteins called endosomal sorting complexes required for transport (ESCRT-0–III) [35,36]. In ESCRT-dependent exosomal formation ESCRT-0 and -I cluster ubiquitylated membrane-associated proteins and lipids into microdomains on the limiting membrane of MVBs. These microdomains recruit soluble cytosolic proteins and RNAs destined for packing as cargoes into exosomes. ESCRT-II and -III then aid in the exosomal budding and fission [37]. 

Alternatively, ESCRT-independent formation involves ceramide subdomain mediated curvature of endosomal membranes and cargo sorting into ILVs [38,39]. Similarly, tetraspanin family members CD9, CD63 and CD81, all of which are highly enriched on the surface of exosomes [40,41,42,43], can mediate ILV biogenesis via clustering of these tetraspanins into microdomains with other tetraspanins, transmembrane proteins and cytosolic proteins that bud from the MVB surface [44], or via formation of CD81 rich domains which induce inward budding [45]. Syntenin 1 has also been shown to be involved in targeting of exosome cargo to endosomal membranes for packaging into ILVs [46], and ADP ribosylation factor 6 (ARF6) can aid ILV budding and sorting of epidermal growth factor receptor (EGFR/HER1) into ILVs, then help traffic EGFR to lysosomes for degradation [47]. 

On the other hand, MVs range between roughly 50 nm to 1300 nm in diameter [29,30,31]. MV formation occurs by direct budding of vesicles from the cell membrane. This process is mediated by ARF6 and RHO family GTPase-dependent rearrangement of the actin cytoskeleton [48,49]. Here, ESCRT-I component tumour susceptibility gene 101 protein (TSG101) traffics to the plasma membrane and interacts with accessory proteins ALG-2- interacting protein X (ALIX) for subsequent cargo sorting and MV release [50]. Furthermore, both neutral and acid sphingomyelinase (N- and A-SMase respectively) modulate downstream activation of receptors that trigger MV release [51,52]. 

Despite their biogenetic differences, the overlapping size ranges and similar proteomic compositions of exosomes and MVs make them difficult to distinguish from one another. This is particularly important as both exosomes and MVs are released simultaneously from their cells of origin into the same extracellular environment, forming an extensively heterogeneous EV population. Consequently, the selective purification of either exosomes or MVs from culture media or biological fluid remains a persistent issue [53]. Therefore, in non-selective studies, or studies where specific markers of subcellular origin cannot be reliably identified, EVs are classed simply according to their size; small EVs (sEVs), i.e., anything smaller than 200 nm, and large EVs (lEVs), i.e., anything larger than 200 nm [54]. lEVs also include oncosomes, a class of EVs first identified by Dolores Di Vizio and her team in prostate cancer. These oversized EVs range from roughly 0.5–5 μm in diameter and have so far only been detected in cancer cell secretomes [55,56].

## 3. The EGFR Family

The receptors of the HER family show more than 80% structural homology and are located on the basolateral membrane of epithelial tissues derived from ectoderm and mesoderm [57]. 

Overall, they are characterised by a ligand-binding extracellular domain (ECD) comprised of two ligand-binding domains (I and III) and two structural cysteine-rich domains (II and IV) [58,59]; a single-helix transmembrane domain, which is essential for receptor interactions and allosteric activation [60,61,62,63,64]; and an intracellular module where the catalytic activity resides. 

**Figure 2:**
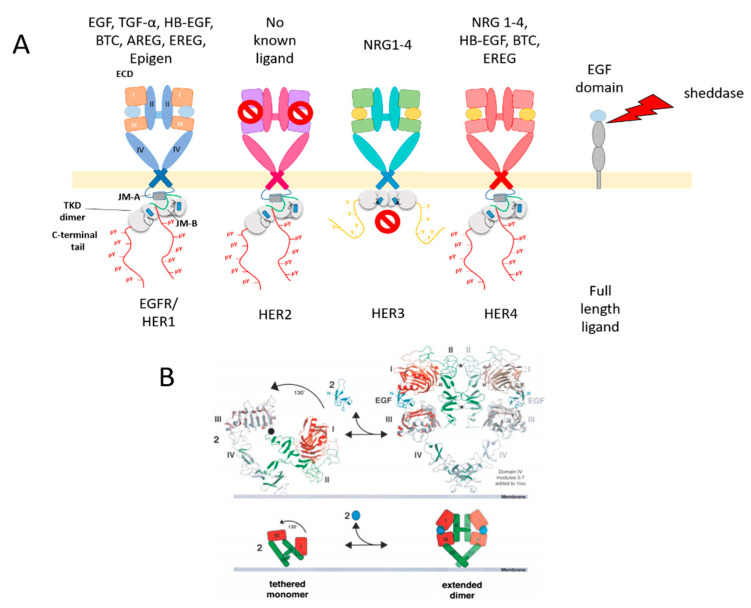
The EGFR family: (**A**) Cartoons of EGFR family homodimers showing the extracellular domain in its back-to-back dimer conformation, bound to ligand where applicable, and the intracellular asymmetric tyrosine kinase dimer, joined by the Transmembrane Domain (TMD). The latter leads to phosphorylation of tyrosines on the C-terminal tail (yellow dots) and recruitment of effectors (red, teal, orange and lime green shapes). The red “no entry” symbols indicate the lack of a known soluble ligand for HER2 and the pseudokinase status of HER3. Also shown is a cartoon of a full-length, transmembrane EGFR family (pro)ligand. (**B**) Molecular model view (above) and cartoon view (below) of the conformational changes involved in ligand-induced dimerisation in the EGFR receptor, adapted from Martin-Fernandez M. et al., (2019).

The intracellular module is comprised of a partially unstructured juxtamembrane domain (JMD) which has regulatory functions and can bind to lipids in the plasma membrane [62,65,66], a tyrosine kinase domain (TKD) and a mostly unstructured c-terminal segment that contains the majority of the phosphorylation sites and plays a regulatory role [67,68] (Figure 2A).

Of the receptors, only EGFR and HER4 [69,70] are fully signalling competent, i.e., capable of both specific binding ligands and catalysing tyrosine phosphorylation for downstream signalling. HER2 does not have a known soluble ligand [71], while HER3 is considered a pseudokinase, as it can bind ATP but its catalytic activity is very low and does not significantly contribute to signalling [72,73]. In spite of this, the kinase domain of HER3 is indispensable for HER2/HER3 signalling [74,75], acting as the activator partner in an asymmetric kinase heterodimer, in which HER2 phosphorylates the C-terminal tail of HER3 [76]. HER2 and HER3 dimers constitute the strongest signalling unit in the HER family and are often co-expressed [77,78]. 

Signalling by HER receptors is induced by allosteric changes induced by ligand binding at the ECD, which release a set of intradomain interactions between subdomains II and IV known as the “tether” and enable dimerisation through an interface comprising domains II and IV [70,79,80], which then leads to allosteric kinase activation [81] (Figure 2B). HER2 constitutes an exception to this rule, as its “untethered” extracellular conformation in absence of ligand mimics that of the EGF-activated EGFR, enabling it to form dimers with the other ligand-bound HERs [82,83]. HER2, however appears to be regulated by a different set of inhibitory interactions between its subdomains I and III, mimicking interactions first discovered in invertebrate EGFR orthologues [84], which appear to be relevant for its heterodimerisation with EGFR [85]. Additionally, the control exerted by the “tether” on EGFR signalling was found to be limited [86] and on cell membrane EGFR is reported to be actually quite flexible, sampling a large conformational space [87].

While oligomerisation and clustering of EGFR family receptors have been reported for a long time [88,89,90,91,92], in recent years, multidisciplinary studies have revealed that these structures are functional and that the EGFR signalling pathway includes not just monomers and dimers, but also a variety of multimeric structures, both in the basal and in the ligand-bound state [93,94]. These might reflect different sensitivities to ligand and/or perform specialised signalling functions [95]. Oligomerisation and alternate dimerisation interfaces are also involved in the signalling of the HER2/HER3 signalling unit, both in unperturbed conditions [76] and in response to targeted HER2 TKIs [96], hinting at common mechanisms of regulation for the whole family. 

## 4. EGFR and Its Ligands on EVs

**Figure 3:**
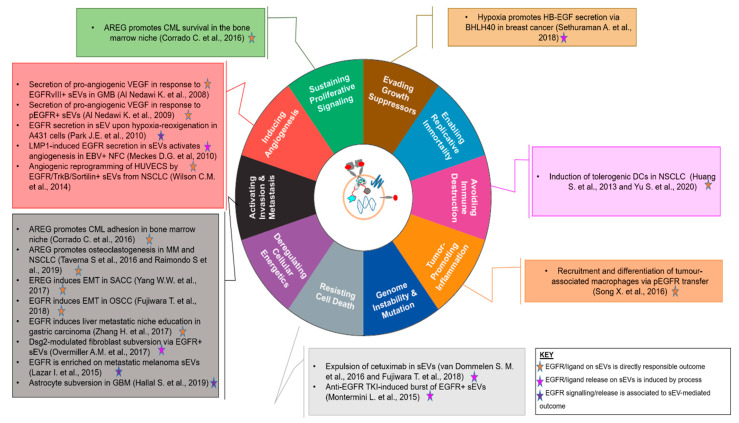
An EGFR-centric view of the involvement of EVs in the Hallmarks of Cancer [97], summarising the key findings for the role of EGFR^+^/Ligand^+^ EVs in the “achievement” of each. The central cartoon schematically depicts a sEV displaying EGFR, a full-length transmembrane EGFR family ligand, a tetraspanin marker and nucleotidic cargo. Findings are denoted by first author and year and colour-coded according to the role played by EGFR and/or its ligands in the biological outcome referenced.

EGFR family members and cognate ligands are expressed by EVs released by neoplastic cells and play a wide variety of roles in enhancing the malignant potential of their parental cells, with a range of effects that covers most if not all of the hallmarks of cancer [97] (Figure 3 and Table 1). 

The expression of EGFR was first identified in 2008 at protein and mRNA level in sEVs derived from cultured EGFR-positive cancer cell lines and clinical samples [31,98,99]. In this context, Al-Nedawi et al. found that EGFR and its glioblastoma deletion mutant isoform EGFRvIII could be packaged in EVs, where they could appear in their phosphorylated state, and that they were functional when internalised by recipient cells, increasing downstream MAPK signalling and the expression of oncogenic factors. This process was termed horizontal oncogene transmission [31] and has later been observed also in a variety of other processes involving wt or mutant EGFR, including metastatic niche establishment and immune suppression [100,101,102].

### EGFR Ligands on EVs

EGFR can bind to seven cognate ligands, which display different affinities, potency and induction of signalling pathways [113,114,115,116,117]. They are synthesised as high molecular weight transmembrane pro-ligands, which are displayed on the surface of parental cells and then released as soluble fragments in response to various stimuli thanks to the action of ADAM proteases [118], a class of proteins commonly present in the EV cargo [119] (see Figure 2A).

Full-length N-term-out functional EGFR ligands Amphiregulin (AREG), heparin binding EGF (HB-EGF) and transforming growth factor alpha (TGF)-α were first identified in sEVs derived from a panel of breast and colorectal cancer cell lines using fluorescence-activated vesicle sorting (FAVS) [120]. AREG was identified as particularly resistant to degradation when displayed on sEVs and as the strongest inducer of invasiveness among the three ligands in the panel of cell lines, perhaps owing to its high stoichiometry (24 ± 7.6 copies/sEV) [120]. AREG is a low-affinity EGFR ligand which has physiological and pathological roles, including in the progression of various types of cancer [121]. Its pleiotropic effects would explain why it emerged in as a major player in sEV-mediated signalling.

AREG expressed on sEVs derived from Chronic Myeloid Leukaemia (CML) cell lines or patient serum is able to activate EGFR on stromal cells in an in vitro model of a bone marrow (BM) niche. Stromal cells EGFR activation induces via SNAIL pathway the secretion of IL-8 and MMP-9, which promote CML cell survival, as well as expression of Annexin 2 which promotes CML cell adhesion and homing to the niche. The significance of its role in niche regulation is highlighted by the fact that AREG expression on CML sEVs is correlated to aggressiveness and chemoresistance in patients [103].

The role of EGFR signalling, and in particular of EGFR ligands, in osteoclastogenesis is known from breast cancer co-culture models and non-small cell lung cancer (NSCLC) models, where EGFR inhibition with TKIs was shown to prevent osteoclast differentiation [122,123,124]. Recent studies have highlighted a key role of EVs in this pathogenesis mechanism, both in bone metastatic NSCLC, where increased plasma levels of AREG are correlated with poor prognosis [105], and in multiple myeloma [104], a highly osteoclastogenic disease where AREG is a known tumour growth factor [125]. In both cases, AREG displayed on EVs is able to induce the in vitro differentiation of pre-osteoclasts, with reduction in OPG levels, increase in RANKL expression and secretion of osteolytic enzymes such as MMP-9 and TRAP [104,105].

Besides inducing pro-tumoural changes in bystander stromal cells, EGFR ligands can also induce cancer progression by acting on their own parental cells. Salivary Adenoid Cystic Carcinoma (SACC) is a type of head and neck cancer that presents a poor prognosis and a penchant for metastasising to the lungs. In this tumour type, expression of low-affinity, high-efficiency EGFR ligand epiregulin (EREG) [126] is associated with poor outcomes and induces EMT, with consequent increases in angiogenic signalling [106]. While previous studies had already highlighted the role of EREG in the induction of SACC metastasis to the lung via the activation of EMT pathways involving the EGFR-Snail/Slug axis [127], a recent study demonstrates that the vehicle for EREG transport to target cells is constituted by sEVs, and that these are responsible for morphological changes in bystander endothelial cells and the induction of angiogenesis [106].

The secretion of EVs carrying EGFR ligands can occur through stimulation. Cationic lipids induce the release of EGF^+^ EVs from melanoma cell lines [128], while breast cancer cells are known to respond to hypoxia by secreting increased numbers of sEVs, a mechanism that favours the secretion of EGFR ligands such as HB-EGF [129]. Studies using murine models of breast cancer have identified BHLHE40, a transcriptional target of HIF-1α, which is itself a transcriptional regulator, as an inducer of several cytokines and growth factors in response to hypoxia. BHLHE40 acts as a transcriptional activator of HB-EGF by binding to its promoter and preventing HDAC1/2 from repressing its transcription. Upregulated HB-EGF is then packaged on upregulated sEVs, which induce survival and migration in recipient cells. The clinical relevance of this mechanism seems supported by the positive correlation between HB-EGF and BHLHE40 expression in TGA datasets [107].

However, why would cancer cells invest in releasing membrane-bound vesicles, when they could just secrete soluble ligands? One explanation is that EV-incorporated transmembrane ligands seem to be much more efficient in terms of concentration and length of induced signalling than recombinant soluble ligands in inducing downstream effects in recipient cells [130]. 

Signalling by full-length, transmembrane ligands to receptors expressed in trans on neighbouring cells, a process called juxtacrine signalling, has been identified since the early 1990s as an independent function for many EGFR ligands, including TGF-α [131], EGF [132], HB-EGF [133], and AREG [134].

More recently, recognising the importance of this mode of signalling, Higginbotham et al. [120] suggested using the term ExTRAcrine (Exosomal Targeted Receptor Activation) for the signalling by transmembrane EGFR ligands displayed on sEVs, a mode which straddles the divide between endocrine for the communication range and juxtacrine, for the binding mode, which is key in determining signalling properties. Owing to their small size, EVs act as a single-point, directional delivery mechanism for the activation of EGFR family receptors, one which concentrates a high density of ligands in a small space. Time-resolved fluorescence experiments have shown not only that point-stimulation of EGFR can be propagated to the entire cell [135], especially if the recipient cell overexpresses EGFR or a local increase in receptor density is created [136], and that multivalency amplifies EGFR signalling, but also that clustering of EGF-displaying particles can lead to even higher signalling amplification [137]. It might also be a matter of fine-tuning the extent of clustering on a spatial level: other investigations have shown that small-scale clusters of EGFR lead to higher phosphorylation and signalling than large-scale clustering [138].

Thus, it seems that there is a clear advantage in being able to deliver a mitogenic signal in well-packaged quanta instead of in a continuous trickle.

## 5. EVs Expressing EGFR Shape the Tumour Microenvironment

The tumour microenvironment (TME) is composed by the tumour cells, stromal cells including fibroblasts, blood and lymph vessels, immune cells, the extracellular matrix (ECM), cytokines, RNAs and small organelles such as EVs. The interplay of tumour cells with their TME influences tumourigenesis, progression and metastasis, drug resistance and has an impact on patient prognosis [139].

Among the soluble factors, EVs play major roles in the complex tumour–TME relationship as, depending on their parental cells, they can support intercellular communication, contributing to the acquisition of functional features by TME components, such as immune cells and endothelial cells, leading to immune suppression and angiogenesis, to mention only some of the effects detailed below.

EVs have different ways of shaping their microenvironment and distant sites depending on their type of interaction with the recipient cells. The factors and mechanisms that mediate EV cargo transfer as well as those that determine how EVs “choose” their target cells are still yet to be fully understood. However, these processes seem to depend on the cells that the EVs originate from, the phenotype and origin of recipient cells and the subsequent effects elicited by the EVs on their recipients [140,141].

Additionally, it remains unclear which moieties on the cell membrane may promote or inhibit EV docking based on their expression level, protein structure or other features like proximity. Another important question is if EVs travelling as single vesicle or in droves may have different effects during their interaction with target cells. However, ultimately the general mechanisms of uptake and intercellular trafficking of different EV subpopulations, such that EV cargoes can evoke their intended effects, are likely to be shared [141]. Major mechanisms of EV uptake include phagocytosis, macro- and micropinocytosis, clathrin mediated endocytosis (CME) and clathrin-independent endocytosis, all of which and more have been reviewed elsewhere [141,142,143].

With these outstanding questions in mind, in the following sections we will expose the shaping activity of EVs in an EGFR context.

### 5.1. Proangiogenic Signalling

Since the very beginning of this investigation field, the induction of angiogenesis downstream of EV uptake by bystander cells has been a major concern. 

Early on, the uptake of EGFRvIII sEVs by human glioblastoma cell line U373 has been demonstrated to increase their secretion of the proangiogenic factor VEGF [31], which then induces angiogenic responses in neighbouring endothelial cells. Proangiogenic effects have been demonstrated also for activated EGFR (pEGFR) displayed on sEVs originating from other cancer cell lines, including A431, A549 and DLD-1, which, in turn, can induce the activation of MAPK and Akt signalling and initiate an autocrine VEGF signalling loop in recipient endothelial cells. The ability of sEVs to transfer pEGFR from parental cancer cells to endothelial cells confers them an advantage in terms of elicitation of proangiogenic responses and tumour growth, which can be reversed in both cell cultures and mice xenografts by inhibiting the uptake of sEVs with Annexin V [108].

Subsequent proteomics studies have confirmed that hypoxia stimulates EGFR-overexpressing A431 cells to secrete both soluble proangiogenic factors and sEVs loaded with proangiogenic proteins. EGFR itself is increased on the surface of sEVs produced by cells upon a hypoxia-reoxygenation cycle [144], which hints at its role as an indirect inducer of angiogenesis. 

Even when EGFR is not the master oncogene, its oncogenic role in the induction of downstream effects through sEVs cannot be overlooked. In EBV-induced nasopharyngeal carcinoma, viral protein LMP1 induces an increase in cellular EGFR expression. Overexpressed EGFR is packaged into sEVs together with LMP1, and its packaging in EVs is increased by LMP1 expression. LMP1^+^/EGFR^+^ sEVs are also positive for PI3K and, when taken up by bystander HUVEC cells, they increase the activation of both Erk and Akt downstream pathways, influencing their angiogenic potential [109].

Finally, in human NSCLC cell line A549, EGFR is expressed on sEVs in a trimeric complex with receptors TrkB and sortilin [110], the former of which is another RTK that can cross-talk with EGFR family members in various cellular contexts [145,146,147], and the latter of which has been found to regulate EGFR signalling in lung cancer by promoting its internalisation [148] and is necessary for sEV release from the MVB pathway [110]. HUVEC cells exposed to sEVs displaying this triple EGFR/TkrB/sortilin complex acquire enhanced MAPK and Akt pathway signalling, enhanced migratory capabilities and increased secretion of soluble proangiogenic factors. These last findings highlight the importance of the identification of specific signalling complexes to understand the functional heterogeneity of EGFR-containing sEVs, an area of research that is not yet sufficiently explored.

### 5.2. Epithelial–Mesenchymal Transition (EMT) Program

Apart from conferring tumour cells an advantage in survival by directly inducing proangiogenic pathways, sEVs are a major player in the induction of EMT and in the propagation of the EMT-mediated effects to the microenvironment in terms of angiogenesis induction, niche subversion and invasion [149]. In addition, EGFR signalling is also heavily involved in EMT processes. On one hand, in a physiological context, EGFR is involved in epithelial wound healing, which includes a partial EMT process through a pathway that includes Erk5 and the Slug transcription factor [150]. EGFR signalling and wound healing appear to be strictly associated with sEVs secretion in renal tubule wound healing, where EGFR signalling seems to decrease sEV output, and vice versa [151].

On the other hand, EGFR can induce EMT in several tumourigenesis contexts, either driving it entirely through Ras or Akt signalling and induction of transcriptional activity [152], or acting downstream of EMT-initiating transforming growth factor beta (TGF)-β signalling [153]. EMT is also involved in resistance to EGFR-targeted therapeutics in NSCLC via activation of Axl in response to EGFR inhibition [154]. This makes the study of the convolution between EMT processes and EGFR signalling even more pivotal for the development of effective therapies.

In HSC-3, an Oral Squamous Cell Carcinoma (OSCC) cell line that expresses high levels of EGFR, exposure to nicotine increases the secretion of EGF, which is a major driver of EMT in this cellular context. EGF signalling then induces the enrichment of EGFR protein in HSC-3 sEVs in a way that cannot be countered by anti-EGFR antibody cetuximab [155], a therapeutic agent in clinical use for colorectal tumours and other EGFR-driven clinical entities which binds to the extracellular domain of EGFR and blocks ligand binding and signalling [156]. HSC-3-derived EGFR^+^ sEVs, in turn, induce EMT markers such as increased vimentin and spindle-like shapes in bystander epithelial cells. This effect is increased by OSCC cell pretreatment with EGFR ligand EGF, which increases the production of EGFR^+^/CD9^+^ sEVs even independently of nicotine. Cetuximab, in this case, is able to block sEV internalisation and inhibit sEV-driven EMT in bystander cells [157]. It is worth noting that metastatic sublines of HSC-3 express higher levels of EGFR in their sEVs in association with heat shock proteins even without exposure to stimuli [158].

### 5.3. Immune Modulation

Immune modulation is a key tactic for tumour cells as it allows them to escape from immune clearance and receive support for growth, dissemination and resistance to therapy from tumour-associated immune cell populations, thereby becoming even more aggressive and deadly (see, for example, in [159,160,161]). Mechanisms allowing tumour cells to avoid immune recognition and escape from immune clearance are continuing to emerge and the different signalling pathways controlling this phenomenon are interconnected with those controlling angiogenesis, proliferation, survival, senescence and metabolism. Communication via vesicles by tumour cells is a key factor in this context, as shown by the numerous studies demonstrating how tumour EVs contribute to generate an immune suppressive state through the induction and support of immune suppressive cells, of which the major populations are represented by T regulatory cells (Tregs) and the myeloid-derived suppressor cells (MDSCs) [162,163,164,165,166]. Their accumulation at tumour sites but also at a systemic level represents a negative prognosis, especially in patients affected by different cancer types in their advanced stages [167,168]. Additionally, MDSCs and Tregs can hinder effective cancer therapy, including immunotherapy with immune checkpoint inhibitors (ICIs) [169,170]. 

Copious evidence has documented that tumour-derived EVs contribute directly to the generation of suppressive immune cells, and this occurs through different mechanisms including transfer of genetic and protein material, and direct signalling [171,172,173]. In turn, suppressive immune cells can release their own EVs propagating immune suppressive effects at a cellular level [174]. 

The involvement of EGFR signalling in the induction of pro-tumour immune tolerance has been characterised particularly well for NSCLC, where it has been demonstrated that EGFR^+^ sEVs can induce differentiation of tolerogenic dendritic cells (DCs), which suppress the anti-cancer immune response [111]. In the case of cells expressing EGFR-del-19 mutant, this effect has been traced back directly to oncogene transmission to infiltrating DCs through sEV uptake. These subverted DCs were then responsible for repressing T cell response against tumoural cells, and resulted in an overall decrease in intratumoural CD8^+^ T cells [102]. In addition, sEVs displaying EGFR or HER2 have also been found to be involved in the recruitment and differentiation of tumour-associated macrophages (TAMs). In in vitro experiments, monocytes were stimulated to survive and differentiate within a simulated inflammatory microenvironment by sEVs derived from a panel of cancer cell lines from different histotypes. This survival signalling resulted from the activation of MAPK and Ras pathways via the horizontal transfer of phosphorylated EGFR or HER2 [101]. 

In summary, incorporation of EGFR in EVs appears to have major relevance in EV-mediated immune modulation with wide ranging consequences impacting also current therapeutic approaches in cancer clinical practice. Unravelling the underlying mechanisms will help targeting the coexisting pathways to enhance therapeutic efficacy.

### 5.4. Tumour Terraforming: Primary Tumour, Pre-Metastatic and Metastatic Niche Education

First advanced in 1899, Paget’s seed and soil hypothesis regarding the non-random distribution of metastases has since been confirmed by a wealth of data regarding the interactions between tumoural cells and the stroma at the site of the metastasis [175]. 

A series of seminal studies has highlighted that sEVs are able to educate the pre-metastatic niche (PMN), preparing the terrain for the arrival of cancer cells. sEVs are able to influence vascular permeability and the expression of inflammation and ECM proteins at distal sites from the primary tumour [176], and determine the organ tropism and metastatic potential of cancer cells injected in immunodeficient mice [176,177]. Their specific targeting, down to a single cell type in the complex microenvironment of a target organ, and action is due to the selective enrichment of specific integrin isoforms [177] or of cytokines and growth factors specific to microenvironmental components of the target metastatic site, which induce signalling loops favourable to the establishment of cancer cells [178].

PMN education via sEV has been identified also in EGFR-dependent context. EGFR expressed on gastric carcinoma sEVs is transferred to primary liver cells, where it colocalises with E-Cadherin and induces a decrease in the expression of miR-26a and miR-26b. These miRNAs are responsible for the post-transcriptional regulation of hepatocyte growth factor (HGF) mRNA translation through their binding to specific sequences in its 3′UTR, so the net effect of the horizontal transfer of EGFR to liver cells is an increase in HGF secretion in the liver microenvironment, which creates the ideal conditions for the establishment of gastric carcinoma cells, as they express high levels of the HGF receptors c-Met on their plasma membrane [100].

In EGFR-overexpressing HNSCC cell line A431, instead, the desmosomal cadherin desmoglein-2 (Dsg2), which can activate EGFR through a c-Src/Caveolin-1-dependent pathway [179], has been found to be expressed on sEVs and regulate the packaging of both EGFR and c-Src. sEVs from cells overexpressing Dsg2, which contain also increased EGFR, can induce phosphorylation of EGFR and downstream signalling pathways in normal human fibroblasts [112]. This could be a mechanism by which epithelial tumours subvert fibroblasts in the TME into a tumour-supporting role, creating a favourable niche where the tumour can become locally invasive. 

Glioblastoma-derived sEVs have been shown to reprogram the main constituent of their microenvironment, astrocytes, to serve in supporting role for tumour growth [180]. This subversion, whereby astrocytes undergo changes in their proteome that favour the secretion of angiogenesis factors and the degradation of basement membranes to aid glioblastoma invasion, is achieved through the activation of MYC and p53 signalling pathways in recipient cells, where EGFR has been identified as one of the predicted upstream regulators of the network [181]. This seems to indicate that EV-displayed EGFR has a pivotal role in this process, but further studies will be required to confirm it.

EGFR is also part of a set of proteins specifically enriched on metastatic melanoma cell line derived sEVs [182], the internalisation of which by bystander cells increases their migratory potential, hinting at a more general role of EGFR in the establishment of metastasis, perhaps by playing a role akin to that of c-Met [176]. 

It appears that much like c-Met, EGFR has the potential to be a master regulator of the tumour niche, thanks to its capacity for horizontal transfer and its multiple interactions with other RTKs both from its own family and from the wider RTK superfamily (see, for example, in [145,183,184,185,186,187]).

### 5.5. Drug Resistance and Its Transfer to Bystander Cells

The role of sEVs in drug resistance mechanisms has been a focus of research since the early 2000s, when Shedden et al. noted a correlation between the expression of genes belonging to a “vesicle shedding index”, comprising most sEV markers identified until then, and the ability to resist a panel of drugs in the NCI-60 cell line panel [188]. Since then, sEVs have been implicated in drug resistance and its transfer to other cell types in the TME in various tumours. EVs can perform this function through a variety of mechanisms, including the transfer of ABC transporters and other drug efflux molecules to bystander cells [189].

Owing to its pivotal role in many of the major killers in the oncological field, EGFR has been at the centre of major efforts in pharmaceutical development [190], which have led to the development of two main classes of drugs, tyrosine kinase inhibitors (TKIs), such as erlotinib, afatinib or osimertinib, that target the ATP binding pocket of the TKD and inhibit its activity [191] and can bind reversibly or irreversibly to EGFR, and monoclonal antibodies such as cetuximab and pertuzumab, which target the extracellular domain and are able to block ligand binding [156], induce antibody-dependent cell cytotoxicity [192], or induce internalisation and downregulation of target receptors [193]. While some of these targeted treatments have been highly effective in the clinic on selected patients harbouring sensitising mutations, resistance to therapy happens inevitably, through genetic and non-genetic mechanisms [194,195].

Regarding the involvement of EVs in this context, findings seem controversial. Cetuximab treatment of EGFR-overexpressing A431 cell line induces a decrease of the expression of EGFR and phosphorylated EGFR on sEVs. However, cetuximab itself is found displayed on sEVs, a mechanism that could represent a way for cells to get rid of an inconvenient inhibitor by expelling it to the extracellular environment [196]. A different study, instead, concluded that while cetuximab does not seem to influence the global production of sEVs by A431 cells or the packaging of EGFR in sEVs, treatment with irreversible EGFR TKIs CI-1033 and PF-00299804 caused a “burst” in both sEV production and in the packaging of EGFR, including most phosphorylated species, Erk and pErk in sEVs. This burst was also accompanied by a peak in Caspase-3 cleavage and could be mitigated both by inhibition of Caspase-3 and by inhibition of neutral sphingomyelinase, which attests to their “classical” MVB origin [197]. Yet a third study, conducted on OSCC cell line HSC-3, instead, recorded an increase in EGFR expression in sEVs in response to cetuximab. In this case, cetuximab was also found to be robustly secreted on EGFR^+^ sEVs [155].

In the case of glioblastomas, targeted EGFR therapies have demonstrated much promise but relatively little success [198]. Alkylating agent temozolomide (TMZ), which, as of today, is one of the standard chemotherapeutic drugs for this histotype, and HSP90 inhibitor geldanamycin (17AAG) have been demonstrated to reduce both the amount of sEVs produced by the parental cells, and more specifically, the expression of both wild type EGFR and EGFRvIII on sEVs [199]. This seems to hint at a role for both drugs in the fight against EGFR-driven microenvironment subversion.

All of these mechanisms could contribute to the changes in sEV secretion and sEV content in response not just to EGFR-targeted therapies, but also to more generic interventions, by using EGFR as a pivotal point to induce microenvironmental changes.

## 6. EVs As Therapeutic Devices against Tumours

One of the most interesting qualities of EVs is their ability to transfer their cargos to recipient cells. Based on this quality, EVs may present as highly suitable platform for targeted therapeutic delivery. Additionally, EV mediated therapeutic delivery offers numerous advantages: EVs are biocompatible, can be generated from autologous patient cells if required, and have been shown to cross major biological barriers, including the blood-brain barrier [200,201,202]. As such, together these qualities present EVs as a potentially highly fruitful targeted therapeutic delivery platform, thus warranting the substantial attention they have received over the last few decades.

EVs can be engineered to mediate biological functions that are apart from their innate effects. For example, EVs can be loaded with therapeutic RNA or proteins either exogenously, or endogenously in a manner that exploits EV biogenesis machinery [200,201,202,203]. EVs can also be engineered to express specific molecules on their surface that may themselves be therapeutically active, aid in targeting EVs to specific cells, tissues or organs to deliver a therapeutic payload, help EVs fuse with recipient cell membranes or aid endosomal escape following uptake [204].

However, while engineering EVs is necessary to utilise them in the treatment of diseases they may otherwise not have any effect on, EVs do possess innate therapeutic potential in a number of disease contexts. The innate therapeutic benefits of EVs has mainly been explored in connection with EVs derived from cells that express stem cell-like properties, with particular focus on mesenchymal stem cells (MSCs). A recent review has highlighted the significance of MSC-EVs in this context and the multitude of preclinical and clinical studies that are taking place to exploit the innate therapeutic and regenerative benefits of MSC-EVs. In particular, MSC-EVs are being investigated for the treatment of respiratory diseases, including pulmonary hypertension, hepatic diseases including hepatic injury and liver fibrosis, renal diseases such as acute kidney injury, neurological diseases including global cerebral ischemia and traumatic brain injury and spinal cord injury, cardiovascular complications such as myocardial infarction and musculoskeletal disease including arthritis and even bone fractures [205].

Some of the highest profile uses of therapeutic EVs have been in a cancer context. In 2013, Ohno et al. engineered human embryonic kidney 293 (HEK293) cell-derived EVs to contain the tumour supressing miRNA, let-7a. EVs were also engineered to express GE11 peptide on their surfaces, a strong EGFR-targeting ligand. Together these EVs delivered let-7a to EGFR expressing xenograft breast cancer cells in RAG2^−/−^ mice, eliciting a significant (*P* < 0.05) ~60% reduction in tumour size following weekly administration of GE11^+^, let-7a^+^ EVs for four weeks [206]. Their therapeutic action is also due to the fact that these particles are efficiently and rapidly internalised thanks to their ability to act as EGFR ligands [207]. 

Similarly, in another high profile study, EVs derived from immature dendritic cells were engineered to express lysosome-associated membrane glycoprotein 2b (Lamp2b) fused with iRGD, an integrin α-V-targeting peptide. These EVs were subsequently loaded with the small molecule chemotherapeutic doxorubicin by electroporation and administer to female BALB/c nude mice transplanted with MDA-MB-231 breast cancer cells. iRGD^+^, doxorubicin^+^ EVs caused a significant inhibition of tumour growth compared to the control groups (*P* < 0.01). iRGD^+^, doxorubicin^+^ EVs were also significantly less cytotoxic than free doxorubicin (*P* < 0.05) [208]. 

Moreover, unlike the previous two examples, another study exploited the use of a bioactive protein for the treatment of B cell lymphoma and melanoma. K562 multiple myeloma cells were transduced with lentiviral human membrane TRAIL, a protein that binds to death receptors on cancer cells, inducing selective apoptosis. Transduced cells produced TRAIL^+^ EVs and these latter were administered to mice transplanted with SUDHL4 B cell lymphoma cells and INT12 melanoma cells every 48 h. Subsequently, a significant growth inhibition of SUDHL4 (68%) and INT12 (51%) was observed following four intratumour injections [209]. Each of these examples exploits a different EV cargo for the treatment of various cancers, highlighting the utility and flexibility of EVs as therapeutic devices in a cancer context and others. An interesting aspect of EVs as natural therapeutic delivery vehicles lies in their versatile packaging potential: in case of EGFR targeting, EVs could be engineered to express EGFR ligands, and simultaneously, the same EVs could be loaded with different drugs or molecules to potentiate drug activity. For this reason, the use of EVs in the treatment of cancer has been extensively researched. Further significant contributions to this area have been highlighted in a recent review [210]. 

## 7. Exploitation of Circulating EVs As a “Liquid Biopsy” in Tumour Care

In many disease contexts, research is being undertaken to advance diagnostic tools to both complement and replace current diagnostic techniques that may be invasive, painful or otherwise time-consuming. One such avenue of exploration is through noninvasive sampling of bodily fluids for biomarkers of disease known as liquid biopsy. Liquid biopsy can provide real-time feedback on patient condition and response to cellular and molecular therapies [211]. Examples of biomarkers may include circulating cells or DNA [211,212]; however, EVs have also been regarded as biomarkers due to them being potentially released by diseased cells.

Cancer is one such context in which the use of EVs as biomarkers has been rigorously pursued for numerous reasons. First, evidence for the use of EVs in early-stage detection and diagnosis of multiple cancers has been extensively explored [213]. EVs may also help identify cancer subtypes to select the correct treatment [214,215] and assist in determining disease prognosis [216]. Furthermore, while tumour biopsies are often utilised in the diagnostic and monitoring process, non-specific findings during immunohistochemical analysis can elicit false-positive or false-negative diagnoses, resulting in less effective treatment [217]. 

In recent years, a novel analytical tool name ExoScreen that can be used to profile surface proteins on EVs has been developed. This tool can be used to analyse EVs in patient blood and serum without the need for purification. The system has been tested in the context of colorectal cancer and could be used to aid diagnosis. ExoScreen utilises streptavidin-coated “donor” beads that captures a biotinylated antibody specific to a marker of interest on an EV surface. “Donor” conjugates are also incubated with fluorescent “acceptor” beads conjugated to a second antibody that also binds to an epitope on the EV surface. The streptavidin-coated beads are then excited with a 680 nm laser resulting in the release of singlet oxygen. In turn, this excites the fluorescent acceptor beads that fluoresce, but only when the beads are within 200 nm of the donor beads that have bound to the analyte of interest. The complexity of ExoScreen prevents against false positive detection, however also prevents detection of particles larger than 200 nm such as some microvesicles or apoptotic bodies [218].

### EGFR on EVs in Pre-Clinical Studies and in the Clinic

Ever since the discovery that sEVs expressing EGFRvIII from glioblastoma patients can cross the blood brain barrier and be quantified in the serum [219], the EGFR family has garnered notable attention within the EV field, bolstered by its importance as cellular biomarkers.

In pancreatic carcinoma EGFR overexpression is measured in ~30% of cases and cytoplasmic overexpression of EGFR is considered a marker of poor prognosis in the ductal adenocarcinoma subtype [220]. Small studies have evidenced that pancreatic carcinoma cells produce a higher amount of sEVs than their normal counterparts, and that the sEVs display EGFR protein. In addition, while EGFR enrichment on sEVs from pancreatic carcinoma cell lines is still debated [221], EGFR was found to be enriched on sEVs derived from mouse xenografts or from the serum of pancreatic carcinoma patients [222].

A small study using ExoProfile, a novel immunocapture chip, instead identified a significant difference in EGFR expression on plasma-derived sEVs between patients with ovarian carcinoma and normal controls; however, the study only involved a small number (*n* = 15) of patients, so larger studies will be required to confirm the validity of this putative biomarker [223].

In colorectal carcinoma, where EGFR is overexpressed by a variable but high percentage of patients and EGFR targeted therapy is a mainstay of treatment [224], but EGFR expression levels do not necessarily correlate with therapy efficacy, perhaps owing to inconsistencies in immunohistochemical methods [225], sEV-expressed EGFR was identified as part of an A33^+^ colorectal carcinoma-specific proteomics signature [226]; however, no specific claims as to its applicability in the clinic were made.

Glioblastoma is another cancer where EGFR signalling plays a pivotal role in tumorigenesis, via overexpression and the expression of truncation mutants [198]. Due to the difficulty to access the main tumour, sEVs have been at the centre of much attention in glioblastoma research [227].

Using a microfluidic detection chip, magnetic nanoparticle labelling and NMR detection on circulating glioblastoma sEVs, Shao et al. identified a diagnostic signature comprising EGFR, EGFRvIII, PDPN and IDH1 R132H which displays a 90% combined accuracy in distinguishing tumour sEVs from control sEVs. Combining this expression signature and a quantification of vesicle numbers, the authors then profiled the circulating sEVs from patients before and after treatment with alkylating agent temozolomide, and found that there was a significant difference in the index derived by the combined marker between responder patients and non-responders [199]. This study was also conducted on a small (*n* = 24) number of patients, thus the metric needs to be validated in a larger longitudinal cohort before coming into wider clinical use. 

Other groups have instead focused on the detection of mutant and wild-type EGFR overexpression via sEV-packaged RNA in the cerebrospinal fluid of patients, where the mutational status of the parental tumour is accurately represented [228]. This access route can serve as a less invasive way to access molecular genetics information about the tumour. 

In NSCLC, where EGFR overexpression is present in about 50% of patients and has a significant negative prognostic value, while mutations in the TKD which can be targeted with TKIs are present in ~10% of cases [229], the quest to use sEV EGFR as a biomarker has been conducted mainly using antibody array techniques. Multiple members of the EGFR family, including EGFRvIII, which is normally not expressed outside glioblastomas, have been identified in sEVs derived from the plasma of 109 patients [230]. 

A larger study, comprising 431 NSCLC patients and 150 controls, however failed to validate any of the EGFR family receptors expressed on plasma sEVs as a potential diagnostic marker [231]. Likewise, in a parallel study conducted on plasma sEVs from 276 patients, none of the EGFR family proteins identified was found to be a significant predictor of overall survival (OS) after applying the Bonferroni correction for multiple comparisons [232].

These clinical studies, however, failed to take into account vesicle heterogeneity and the importance that different EGFR family complexes might play in determining biological effects.

In spite of these less than promising results, the quest for sEV biomarkers continues with new and more refined strategies. An interesting new development is EXosome-focused Translational Research for Afatinib (EXTRA), a prospective study to identify novel predictive biomarkers and a resistance marker for the TKI afatinib in NSCLC patients that express mutant EGFR [233]. Its biomarker mining strategy leverages big data to correlate clinical efficacy with multi-omic data, the latter of which will be derived from serially collected peripheral blood-derived samples, including plasma, serum and serum-derived sEVs.

Conceivably by combining many sources of information that can capture vesicle heterogeneity, this study will be able to extract useful information about how therapy reflects in sEV secretion in different circumstances.

## 8. Perspectives

As shown by the body of research presented so far, EGFR acts as a major cargo of EVs and participates, with co-expressed proteins and small RNA molecules, in EV uptake and reprogramming of bystander cells in the TME, contributing to most if not all of the hallmarks of cancer (see Figure 3). 

Systematic understanding of the effects of EGFR signalling within these contexts is fast-growing, however it is hampered by EV heterogeneity that extends beyond vesicle biophysical and biogenetic properties. Despite enormous technical improvements, the difficulty of distinguishing EV subtypes is yet to be solved. EV heterogeneity constitutes a major issue faced by the entire EV community in that it presents a steep challenge to our ability to purify, tag, and track specific EV subpopulations on their journey through the parental cell, and out in the microenvironment (reviewed, among others, in [234,235]). 

To cope with resulting heterogeneous descriptions and individual naming of EVs subtypes, the International Society for Extracellular Vesicles (ISEV) has published a “Minimal information for Studies of Extracellular Vesicles-MISEV” 2018 after an extensive survey in the EV scientific community [54]. 

EV heterogeneity is also exhibited by their differing cargos, which may elicit a diverse array of biological effects on physiological or pathological status of the recipient cell, as demonstrated in these exemplary studies. At the gene level, Willms et al. showed that B16F10 melanoma cells release two subpopulations of EVs that can be distinguished based on flotation density. HD-EXO altered the expression of 1116 genes, whereas LD-EXO altered the expression of only 257 genes in recipient endothelial cells [236]. 

In this line at the protein level, Xu et al. (2018) revealed that sEVs and lEVs derived from LIM1863 colon cancer cells displayed 354 and 606 distinctly expressed proteins, respectively, with 256 proteins in common and 350 uniquely enriched proteins in lEVs. Comparatively, lEVs were able to elicit invasive action of recipient fibroblasts, which was roughly three times greater than that induced by sEVs. These differences were attributed to differential enrichment of proteins involved in invasion, migration and cell motility among the two EV populations [237]. 

EV heterogeneity can also cause interesting paradoxes when effects are observed in bulk. Cancer-derived EVs may exhibit opposing effects on immune cells. This can be explained by the existence of EVs exposing different integrin and tetraspanin profiles that correspond to their subsequent immunostimulatory and immunosuppressive activity, thereby modulating these effects by regulating their uptake into specific recipient cells [53].

Given the vast heterogeneity of secreted EVs, the numerous factors that can affect their composition and the plethora of cell sources, so far it has been difficult to clearly attribute any given function to a particular EVs subtype. 

In the case of EGFR, this means that when EGFR expression on sEVs is used as a marker of a specific effect, this does not take into account that the same cell type will likely express several EGFR^+^ subpopulations displaying EGFR complexes with different structure and stoichiometry, as well as distinct sets of post-translational modifications and associated cargo, some of which will not be involved in the process being investigated at all. This is likely to be one of the key reasons why the development of EGFR protein-based biomarkers for liquid biopsies has proven to be so fraught.

This situation is further complicated by EV heterogeneity and the current purification methods that are unable to identify EVs based on their subtype or biogenesis, due to overlapping sizes and continued uncertainty surrounding biogenetic markers [236,238]. 

So-called “Omics” methods are fundamental to reveal the overall composition of the proteomes of EVs, as well as to guide subsequent targeted studies. However, these techniques often lack the spatial and temporal resolution and single-molecule sensitivity which are needed to capture the complexity of EGFR EV biogenesis and signalling, leaving many cardinal questions unanswered.

## 9. Outstanding Questions

In the context of EGFR incorporated in EVs, one key question is how many different EGFR family^+^ subpopulations are secreted by different cancer cells, and under what conditions EGFR and its ligands are enriched in or excluded from EVs. Second, we are also blind to what subtypes of EVs are secreted in response to EGFR signalling and/or are impacted by its inhibition. Immunocapture strategies can do some of the heavy lifting in separating a general EGFR (or ligand)-positive EV subpopulation, but this is likely to be heterogeneous in turn. Furthermore, this first round of detection must be combined with quantitative proteomics and/or FAVS to gain some insight on what other proteins are coexpressed with our chosen target, data which might lead to a future microfluidics-based detection platform for use in pre-clinical and clinical settings.

Differences in the structure and stoichiometry of the EGFR complex, stoichiometry and clustering of ligands, their interactions with other cargo and the presence of other companion molecules will impact the function of EVs in ways we are still not able to quantify, much less control.

Many studies have shown that EGFR^+^ or ligand^+^ EVs display various functional activities once taken up by different types of bystander cells. However, it is almost certain that only some, or even only one, of the EGFR^+^ subpopulations will be involved in each functional activity. The quest to identify which EGFR^+^ EVs are relevant for pro-tumoural activities is likely to be an arduous one, and will rely not just on ever more elegant proteomics experiments, but also by a much-needed “democratisation” as well as improvements in FAVS techniques [234], by the design and development of better EV-labelling strategies, both for bulk and targeted labelling and especially by the development of single-particle and microfluidic detection strategies and devices.

Enlightenment will be brought by high-resolution, live-cell 3D imaging technologies such as multifocal and multiplane microscopy [239,240], 3D structured illumination microscopy [241], multiangle interference microscopy [242], tilted light sheet [243] and lattice light sheet microscopy [244,245], to shed light on the aspects of EV interaction that are still obscure with target cells including timescales of interaction and internalisation. Lattice-light sheet microscopy in particular seems an extremely promising method, as it can combine with single-molecule detection techniques and record single-molecule information about membrane receptor dynamics over the entire cell surface with high spatial and temporal resolution [246], an ideal feature to investigate EV internalisation dynamics. These methods, however, come with their own challenges of high technical difficulty, the requirement for specialised expertise in microscope set up, data analysis and data management and low diffusion among non-specialists. Although, these very challenges open opportunities for interdisciplinary collaboration between the EV community and the advanced light microscopy and CLEM communities, which hopefully will help “democratise” these techniques in the medium- to long-term.

These high-resolution techniques will contribute unveiling the mechanisms of cargo transfer between EVs and target cells, thereby answering open questions like if EVs behave more like synaptic vesicles, promptly releasing their cargo upon fusion with the plasma membrane, or more like enveloped viruses, travelling within a cell’s endocytic pathway before releasing their contents in the cytoplasm.

New and improved fractionation methods and improved knowledge about specific markers of different biogenesis pathways, combined with the other technical improvements outlined above, will also help clarify whether functions of EVs are segregated by cellular origin or whether functions are independent from biogenesis but rather depend on their protein, lipid and RNA complement.

The acquisition of this knowledge will be a key step in the development of therapeutic agents and strategies to block “subversive” signalling by EVs in cancer with the aim of attenuating the angiogenic, metastatic, immunosuppressive and EMT potential of tumours and mitigating or even reversing drug resistance.

## Figures and Tables

**Table 1:** Summary of the effects of EGFR and its ligands when packaged in EVs.

Parental Cancer Type	EV Cargo	Effect	Reference
CML	AREG	Activates bone marrow stromal cells to secrete CML survival factors.	[103]
Multiple Myeloma	AREG	Induces differentiation of pre-osteoclasts	[104]
NSCLC	AREG	Induces differentiation of pre-osteoclasts	[105]
SACC	EREG	Induces EMT in parental SACC cells	[106]
Breast Cancer	HB-EGF	Induces survival and migration of parental cells in response to hypoxia	[107]
GBM	EGFR vIII	Induces secretion of proangiogenic factors by endothelial cells	[108]
Various EGFR^+^ cancer cell lines	pEGFR	Induces secretion of proangiogenic factors by endothelial cells	[108]
EBV^+^ Nasopharyngeal Carcinoma	EGFR	Induces secretion of proangiogenic factors by endothelial cells in response to LMP1 expression	[109]
NSCLC	EGFR/Sortilin/TrKB complex	Induces secretion of proangiogenic factors by endothelial cells	[110]
OSCC	EGFR	Induces EMT in bystander epithelial cells	
NSCLC	EGFR or EGFR del19	Induces differentiation of tolerogenic DCs	[102,111]
Various cell lines	EGFR	Induces survival and differentiation of TAMs	
HCC	EGFR	Represses miR-26a and miR-26b and induces HGF expression	[100]
A431	EGFR	Induction of EGFR signalling in fibroblasts in response to Dsg-2 expression	[112]
